# Dissipative Regenerative Chemiluminescence in Water: A Perylene‐Based Out‐of‐Equilibrium Light‐Emitting System

**DOI:** 10.1002/anie.9467229

**Published:** 2026-08-27

**Authors:** Luca Morgan, Chiara Alberoni, Dario Alessi, Daniel Morales‐Martinez, Sara Bonacchi, Alessandro Aliprandi

**Affiliations:** ^1^ Dipartimento Di Scienze Chimiche Università Degli Studi Di Padova Padova Italy

**Keywords:** chemical energy, chemiluminescence, diimide, dissipative system, hydrogen peroxide, luminophore, luminescence, nanotechnology, perylene

## Abstract

Living systems maintain functional states through the continuous dissipation of chemical energy, a principle that remains a formidable challenge to replicate in synthetic molecular architectures. In such context, while classical chemiluminescence relies on irreversible bond‐breaking events that consume the luminophore, regenerative chemiluminescence (RCL) offers a circular alternative based on electron‐transfer cycles. However, traditional RCL remains constrained by harsh operating conditions and requires isolation of reactive intermediates. Here, we report the development of a water‐soluble Perylene Bisimide (PBI) scaffold capable of undergoing Chemically Fueled Regenerative Chemiluminescence (CFRCL) under mild, dissipative conditions. By strategically tailoring the PBI core we enable a redox cycle driven by mild chemical fuels, sodium dithionite and hydrogen peroxide, in aqueous media. We show that light production can be sustained and pulsed through continuous chemical fueling. These results bridge the gap between redox‐active molecular chemistry and autonomous light‐emitting materials, providing a novel non‐equilibrium analytical tool and bio‐inspired photonic system.

## Graphical abstract

Driven by sodium dithionite and hydrogen peroxide under mild aqueous conditions, a water‐soluble perylene bisimide undergoes reversible electron‐transfer cycles that generate its emissive excited state without dye decomposition. We define this out‐of‐equilibrium system as chemically fueled regenerative chemiluminescence (CFRCL), opening new avenues for autonomous light‐emitting materials and bio‐inspired photonics.

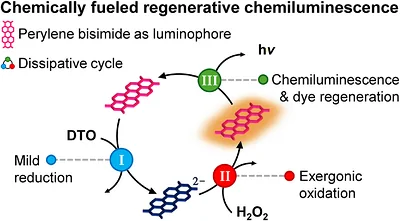

## 1 Introduction

The generation of electronically excited states is fundamental for accessing novel manifolds of chemical reactivity that can be harnessed in photocatalysis and solar energy conversion through electron‐transfer processes occurring from the excited state [1, 2]. A comparatively less explored, but conceptually complementary phenomenon, is the formation of emissive excited states via electron‐transfer reactions in solution. The earliest, and still relatively scarce, examples of this mechanism were reported in the 1960s; however, this area has since received limited attention from the broader chemical community. Within this framework, in 1964 and 1966 Sonntag and Chandross [3, 4] described chemiluminescence arising from the exergonic oxidation of the radical anion of 9,10‐diphenylanthracene DPA^•–^. This radical anion was typically generated by reduction of DPA with metallic sodium or potassium, followed by oxidation using benzoyl peroxide (BPO), or other potent oxidants. Remarkably, the emitting species was unambiguously identified as the neutral DPA molecule in its electronically excited state. This observation led to the definition of a “regenerative chemiluminescence” (RCL) process, in which the luminophore is first reduced and subsequently oxidized to form its excited state; after radiative decay (light emission), the ground‐state luminophore is regenerated and remains available to undergo further chemiluminescent cycles. The overall process can be summarized by the following set of elementary steps:

$$L+\mathrm{ED}\;\to \;L^{-}+ED^{+}$$

$$L^{-}+\mathrm{EA}\;\to \;L^{*}+EA^{-}$$

$$L^{*}\;\to L+\mathrm{hv}$$

with L = generic luminophore, ED = electron donor, and EA = electron acceptor. Notably, this system is fundamentally distinct from classical chemiluminescent processes (e.g., luminol, luciferin, peroxyoxalate) [5, 6, 7, 8, 9, 10], in which the generation of excited states arises from bond‐breaking and bond‐forming events. While such transformations provide a substantial thermodynamic driving force, they also irreversibly convert the initial molecule into a waste product. In contrast, in electron‐transfer‐based RCL, the luminophore can, in principle, participate in multiple excitation–emission cycles. Despite their potential, the chemiluminescent systems reported to date exhibit significant limitations, particularly the severe conditions required to generate their reactive intermediates, often relying on toxic oxidants (PbO_2_ or S_2_O_8_^2−^) [11, 12] or highly reactive reductants, such as alkali metals [3, 4]. Furthermore, these intermediates generally must be isolated prior to the chemiluminescent step to avoid hazardous reactions arising from direct contact between strong oxidizing and powerful reducing agents. The energy required to populate the excited state of a luminophore L can be approximated as described by equation [13].

(1) $$E_{L^{*}/L^{-}}^{0}\;≅\;E_{L/L^{-}}^{0}+E_{0,0}$$

To enable RCL with mild redox agents in a reduction‐oxidation cycle, it is essential to shift the reduction potentials to more positive values while decreasing the E_0,0_ energy. In this context, perylene bisimides (PBIs) emerge as a particularly suitable scaffold, owing to the well‐established tunability of their electrochemical and photophysical properties [14]. PBI derivatives have already found application in organic electronics [15, 16] and photosensitization [17], and have more recently become a cornerstone in the development of synthetic dissipative systems: as shown by Hermans and others, they can be driven far from equilibrium using chemical fuels such as dithionite to control non‐equilibrium self‐assembly, oscillations, and transient functional states [18, 19]. In this way, such systems mirror biological processes, in which a specific state is sustained through the continuous consumption of chemical energy rather than by simple thermodynamic stability.

While this line of research has predominantly focused on structural assembly and disassembly kinetics under dissipative conditions [18, 20, 21, 22, 23], here we apply systems chemistry principles to a different target: chemiexcitation pathways. An electronically excited state is, by definition, an out‐of‐equilibrium species at the single‐molecule level; our aim, however, was to establish a system‐level non‐equilibrium condition, in which the population of excited species is dynamically sustained through the continuous consumption of chemical fuels, rather than simply generated in a single burst.

Building on this idea, we demonstrate that PBIs can act as highly promising RCL luminophores operating under dissipative conditions, a mechanism we designate as CFRCL (Chemically Fueled Regenerative Chemiluminescence). Through strategic tailoring of the PBI architecture, we bridge electron‐transfer‐based emission with systems chemistry design principles. Rather than addressing molecular aggregation, morphology evolution, or assembly kinetics, this work shifts the paradigm toward exploiting a chemical reaction cycle to generate electronically excited states directly, without light irradiation (Figure 1).

**FIGURE 1:**
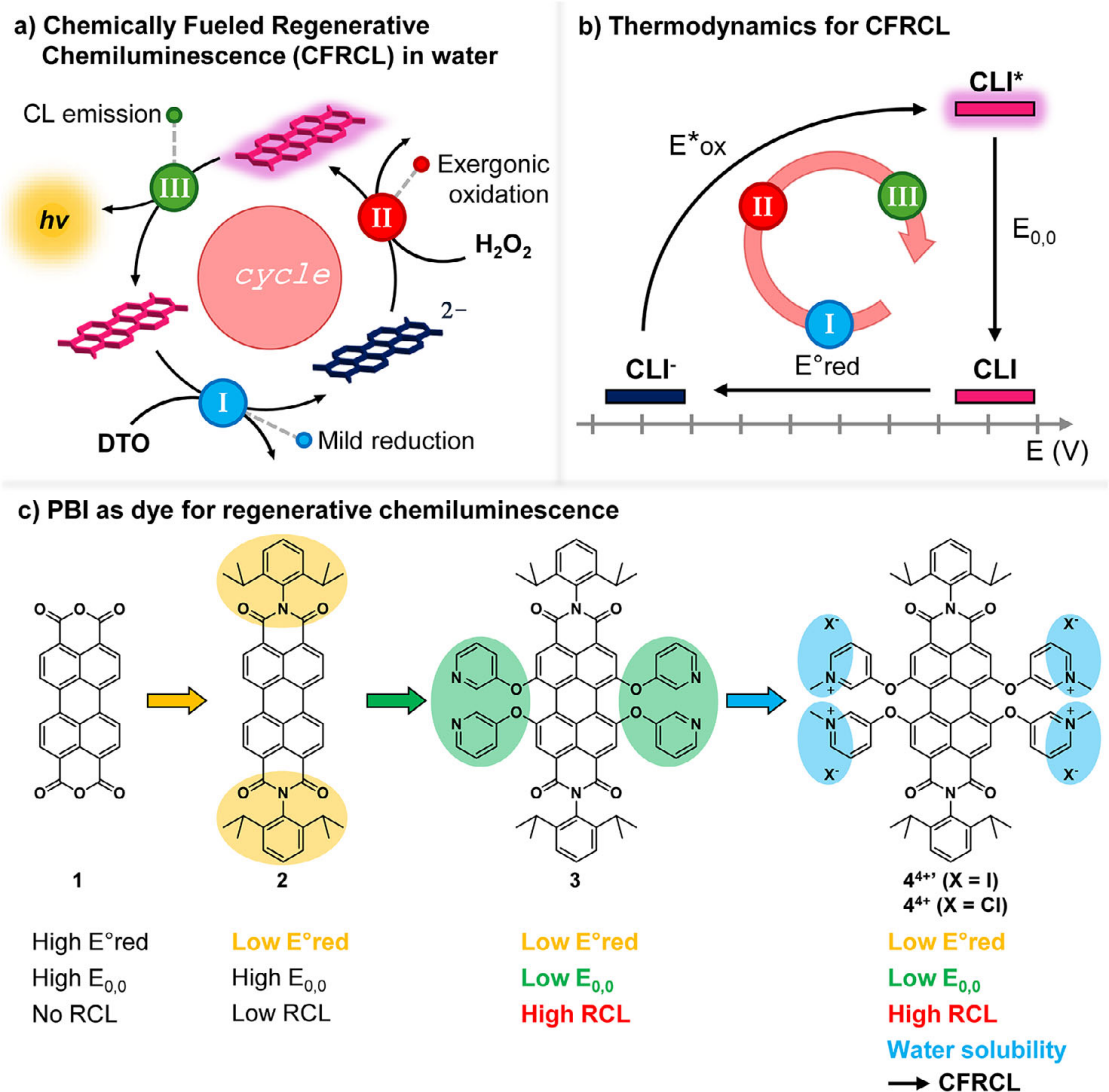
(a) Schematic representation of CFRCL. (b) Representation of the thermodynamics involved into reported out‐of‐equilibrium systems and the dissipative chemiluminescent system reported in this work. (c) Compounds considered in this study.

Notably, light emission can be translated from organic solvents to aqueous media under mild conditions using dithionite and hydrogen peroxide. This represents a key step toward extending the RCL mechanism to biologically relevant environments, where mild reducing agents and strong oxidants naturally coexist, and toward shifting operational control from a thermodynamic regime to kinetic turnover within a dissipative framework. Finally, we examined how the concentrations of the key components influence system behavior, revealing trends that are highly encouraging for potential imaging and analytical applications.

## 2 Results and Discussion

### 2.1 Synthesis and Characterization

Leveraging the synthetic versatility of the perylene scaffold, which allows for the precise tuning of both electrochemical and photophysical properties, compounds **2–4** ^4+^ were synthesized starting from perylene‐3,4,9,10‐tetracarboxylic dianhydride (perylene dianhydride). The synthetic routes followed slightly modified literature procedures [24, 25, 26, 27]. Compound **2** was obtained by the condensation of perylene dianhydride with 2,6‐diisoproprylaniline in imidazole at 140°C. The product was precipitated with 2 M HCl and isolated as an orange solid (57% yield). To achieve bay‐functionalization, perylene dianhydride was first converted to the 1,6,7,12‐tetrachloro intermediate using iodine and chlorosulfonic acid at 65°C. Subsequent nucleophilic aromatic substitution with 3‐hydroxypyridine in DMF yielded compound **3** as a dark violet solid (73% yield). The introduction of four positive charges via N‐methylation with methyl iodide in acetonitrile provided compound **4** ^4+^ **’**. To improve the limited water solubility of the iodide salt, an ion‐exchange resin was employed to obtain compound **4** ^4+^ as a chloride salt (81% yield). Ion‐exchange was confirmed by electrochemical analyses (see Supporting Information).

### 2.2 Electrochemical Properties

The electrochemical behavior of the series was investigated via cyclic voltammetry (CV) in DMF (summarized in Table 1). While the inadequate solubility of compound **1** precluded direct CV measurements, its properties can be inferred from documented soluble derivatives, such as the C10 alkyl‐esterified perylene [28]. This derivative exhibits two reversible monoelectronic reduction waves at –1.30 V and –1.02 V vs. Ag/AgCl, corresponding to the formation of the radical anion and dianion, respectively. The computed LUMO energy for **1** (–2.95 eV) aligns closely with that of the tetraester (–2.70 eV), suggesting that **1** possesses similarly cathodic reduction potentials [29]. Compound **2** likewise showed limited solubility; however, literature reports for this PBI derivative indicate two reversible reductions at –0.85 V and –0.56 V versus Ag/AgCl [30]. This significant anodic shift relative to **1** is consistent with the introduction of the strongly electron‐withdrawing imide groups. The introduction of hydroxypyridine groups at the bay positions in compound **3** further shifted the reduction potentials anodically to –0.56 V and –0.34 V versus Ag/AgCl (Figure S2). This substitution also improved solubility by inhibiting π‐π aggregation through the presence of bulky substituents. Finally, the quaternary ammonium groups in compound **4** ^4+^ resulted in a further anodic shift. Notably, in aqueous media, the two reduction processes for **4** ^4+^ merge into a single wave at –0.19 V versus Ag/AgCl (Figure S3), a phenomenon previously reported for other cationic water‐soluble PBIs [30, 31].

**TABLE 1:** Electrochemical and photophysical properties of the investigated compounds. Compounds **1** – **3** were studied in DMF, compound **4** ^4+^ was studied in deionized H_2_O [30].

Compound	Ered (V vs Ag/AgCl)	*λ*_Abs_, max (nm)	*λ*_Em_, max (nm)	PLQY (%)
**1**	—	455, 483, 517	531, 571	38
**2**	−0.80*; −0.51*	484, 517	535, 574	45
**3**	−0.56; −0.34	437, 525, 563	601	79
**4** ^4+^	−0.19	416, 508, 542	591	59

*Literature data. [30]

### 2.3 Photophysical Properties

Photophysical data of compounds **1**–**4** ^4+^ are summarized in Table 1 (see Figures S5–S14). Compound **1** exhibits the characteristic vibronic structure of the perylene core, with absorption maxima at 455, 482, and 517 nm and fluorescence peaks at 531 and 571 nm (PLQY < 40%). The E_0, 0_ value, determined from the intersection of absorption and emission spectra, was 2.37 eV. Conversion into the bisimide, compound **2**, results in minimal photophysical changes, with absorption at 484 and 517 nm and emission at 535 and 574 nm (E_0, 0_ = 2.35 eV, PLQY = 45%) [32]. In contrast, bay‐functionalization in compound **3** induces a significant bathochromic shift. Its absorption spectrum displays maxima at 437, 525, and 563 nm, with a bright emission at 601 nm and a high PLQY of 79% (E_0, 0_ = 2.13 eV). The PLQY and photoluminescence of compound **3** were also investigated in various DMF/H_2_O mixtures (Figure S13). At high ratios of DMF, PLQY of compound **3** remains elevated and constant, above 75%. For DMF/water ratios below 25%, a pronounced decrease of PLQY was observed, culminating in complete quenching in pure water (100% H_2_O). This behavior can be attributed to aggregation‐caused quenching, which is characteristic of PBI scaffolds [32]. Furthermore, these measurements revealed a bathochromic shift of the emission of compound **3** with increasing water content, from 602 nm in pure DMF (100% DMF) to 614 nm in a 25% DMF/75% H_2_O mixture. Regarding compound **4^4+^**, although the methylation of the pyridine rings causes a slight hypsochromic shift relative to **3** (ca. Δ*λ* = 20 nm), it maintains a structured absorption (maxima at 416, 508, and 542 nm) and bright emission at 591 nm (PLQY = 59%, E_0, 0_ = 2.19 eV) similar to its precursor **3**. The reduction in PLQY observed when progressing from compound **3** to compound **4^4+^** could be ascribed to the increased electron‐withdrawing character of the bay pyridines due to quaternization, accelerating non‐radiative decay. Moreover, measurements of its PLQY in various H_2_O/DMF mixtures (Figure S14), reveal an approximately constant PLQY over H_2_O/DMF volume ratios from 3:4 to 1:4, with a pronounced decrease only in pure DMF (100% DMF). The latter can be attributed to aggregation‐caused quenching mechanism of the highly charged PBI derivative in the less polar solvent. In addition, the photoluminescence spectra, showed a slight hypsochromic shift with increasing DMF content (*λ*_em, max_ = 580 nm at 75% DMF).

### 2.4 Regenerative Chemiluminescence With PBI Derivatives

PBI derivatives were evaluated as luminophores to demonstrate the feasibility of a reduction‐oxidation RCL cycle utilizing mild redox fuels. Sodium dithionite (DTO) was selected for the initial reduction step due to its established efficacy in generating PBI radical anions and dianions [18, 19, 23, 31, 33, 34]. Given that DMF was the primary solvent for compounds **1–3**, dithionite was pre‐dissolved in deionized water to ensure rapid, homogeneous mixing upon addition to the organic phase (final H_2_O/DMF ratio 1:10). For the subsequent oxidation step, hydrogen peroxide (H_2_O_2_) was chosen. Although atmospheric oxygen can effectively restore the neutral form of PBIs, this pathway lacks the exergonicity required to populate the excited state. In contrast, H_2_O_2_ provides a significant thermodynamic driving force, capable of exciting the luminophores upon oxidation of the reduced species.

The investigation began with the commercially available compound **1**. As noted in the electrochemical characterization, **1** exhibits the most cathodically shifted reduction potentials in the series, rendering it incompatible with reduction by dithionite. UV–vis absorption spectroscopy confirmed this inertness: the addition of 100 equivalents of DTO to a degassed solution of **1** (5·10^−6^ M in DMF) induced no spectral changes (Figure S15), precluding its participation in an RCL cycle.

In contrast, the structural modifications in compound **2** allow for a rapid chemical reduction with dithionite. Upon addition of 100 eq. of aqueous DTO, the solution color changed immediately from orange to violet (see Figure S29 for representative photographs illustrating the variations in absorption and fluorescence observed for the solutions.). UV–vis monitoring showed the disappearance of the neutral PBI absorption bands and the emergence of intense new peaks at 510, 549, 621, and 693 nm (Figure S16, orange line), characteristic of the dianionic species **2** ^2−^ [30, 31, 33]. However, subsequent addition of H_2_O_2_ resulted in an unstructured absorption band centered at 501 nm (Figure S16, yellow line) rather than the recovery of the original dye. This indicates that **2** undergoes irreversible degradation after a single redox cycle. This observation was corroborated by the appearance of broad signals in the NMR spectrum of the RCL product, incompatible with compound **2** (See Figure S17). Preliminary RCL tests on **2** (1 mM in DMF) yielded only negligible light emission (Figure S18). Even at lower concentrations (1·10^−5^ M) designed to prevent π‐π stacking‐induced quenching, no detectable luminescence was observed upon injection into the H_2_O_2_ solution (Figure S19). These results underscore that the efficient regeneration of the starting luminophore is a prerequisite for successful RCL; here, the chemical energy of oxidation is likely dissipated through the formation of non‐emissive degradation by‐products.

Compound **3**, featuring bulky bay substituents, was designed to reduce aggregation tendency while enhancing chemical stability. Upon treatment with 100 eq. of DTO, a bathochromic shift to a peak at 686 nm was observed (Figure 2a). Crucially, the addition of H_2_O_2_ led to the complete recovery of the neutral species, confirming the robustness of the scaffold. To identify the reduced intermediates, spectroelectrochemical analysis was performed (Figure 2b). The radical anion **3^•^** ^−^ (generated at –0.36 V) displays an intense band at 777 nm and a weaker NIR band at 964 nm. The dianion **3** ^2−^ (generated at –0.69 V) exhibits a blue‐shifted, sharp band at 690 nm. The close match between the spectroelectrochemical profile of **3** ^2−^ and the chemically reduced intermediate (686 nm) confirms the formation of the dianionic state.

**FIGURE 2:**
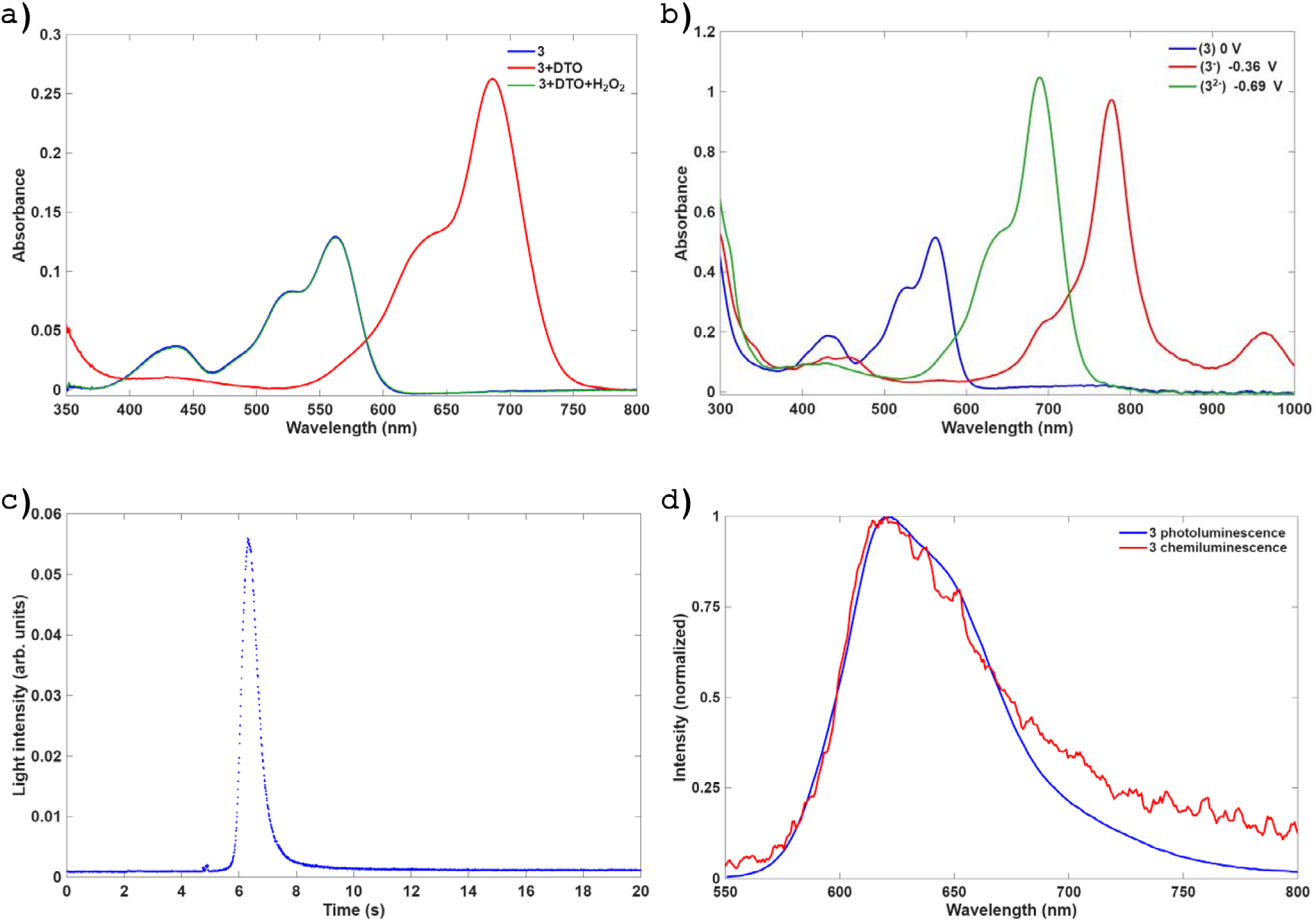
(a) Absorption spectra of **3** (degassed solution) (blue curve), **3** ^2−^ (reduction with 100 eq. of aqueous DTO) (red curve), **3** (after H_2_O_2_ addition to **3** ^2−^) (green curve). [**3**] = 5·10^−6^ M in DMF. (b) Absorption spectra of: **3**, E_pulse_ versus Ag/AgCl = 0 V (blue curve); **3** ^•−^, E_pulse_ versus Ag/AgCl = −0.36 V (red curve); **3** ^2−^, E_pulse_ versus Ag/AgCl = −0.69 V (green curve). Constant pulse electrolysis for 300 s, GC working electrode (custom), Pt wire as CE, Ag wire pseudo RE, [**3**] = 0.1 mM, supporting electrolyte: TBAPF_6_ 0,2 M, solvent: DMF. (c) Light intensity versus time plot of **3**. [**3**] = 1 mM, [H_2_O_2_] = 1 M, solvent: DMF. PMT gain at 100 equipped with optical fiber. (d) Comparison of the emission spectra of **3**: photoluminescence (blue curve), regenerative chemiluminescence (red curve).

When a solution of **3** ^2−^ (1 mM in DMF) was injected into H_2_O_2_ (1 M), a strong chemiluminescence signal was recorded (Figure 2c). The CL emission spectrum matches the steady‐state photoluminescence (PL) of **3** (Figure 2d), identifying it as the emitting species. The observed discrepancy between the PL and CL maxima can be attributed to autoabsorption at concentrations in the mM range (see Figure S20). This demonstrates that rational molecular design can transform the PBI core into an efficient RCL luminophore triggered by mild fuels.

Building on these results, we evaluated compound **4** ^4+^ as a water‐soluble RCL dye. Operating in aqueous media is a significant milestone for developing RCL systems capable of functioning in biological environments, where mild reductants and reactive oxygen species (ROS) coexist.

Spectroscopic analysis of **4** ^4+^ (5·10^−6^ M in water) showed a rapid color change upon DTO addition, with the emergence of a peak at 658 nm (Figure 3a). Spectroelectrochemical data (Figure 3b) confirmed this species as the dication **4** ^2+^. Consistent with the stability of its precursor, the addition of H_2_O_2_ immediately restored the original absorption spectrum of **4** ^4+^ (Figure 3a).

**FIGURE 3:**
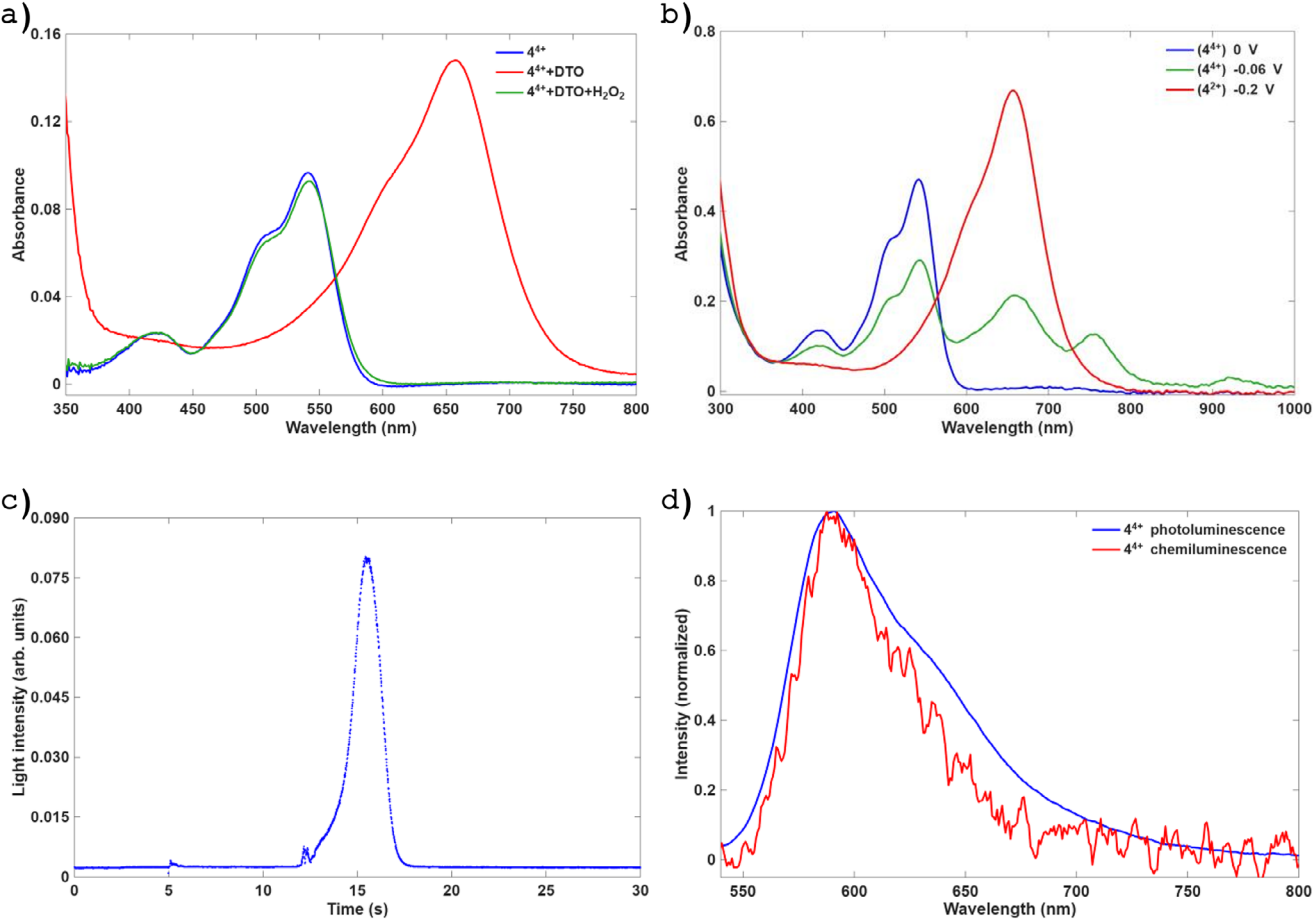
(a) Absorption spectra of **4** ^4+^ (degassed solution) (blue curve), **4** ^2+^ (reduction with 100 eq. of aqueous DTO) (red curve), **4** ^4+^ (after H_2_O_2_ addition to **4** ^2+^) (green curve). [**4** ^4+^] = 5·10^−6^ M in water. (b) Absorption spectra of: **4** ^4+^, E_pulse_ versus Ag/AgCl = 0 V (blue curve); “**4** ^3+^”, E_pulse_ versus Ag/AgCl = −0.14 V (red curve); **4** ^2+^, E_pulse_ versus Ag/AgCl = −0.33 V (green curve). Constant pulse electrolysis for 300 s, GC working electrode (custom), Pt wire as CE, Ag wire pseudo RE, [**4** ^4+^] = 0.1 mM in PBS. (c) Light intensity versus time plot of **4** ^4+^. [**4** ^4+^] = 1 mM, [H_2_O_2_] = 1 M, solvent: water. PMT gain at 100 equipped with optical fiber. (d) Comparison of the emission spectra of **4** ^4+^: photoluminescence (blue curve), regenerative chemiluminescence (red curve).

RCL experiments in distilled water yielded intense light emission sustained for approximately 5 seconds (Figure 3c). The identity of the emitter was confirmed by the overlap of the CL and PL spectra (Figure 3d). These findings demonstrate that an RCL‐inert PBI scaffold can be re‐engineered through targeted functionalization into a highly efficient, water‐soluble system driven by a mild, reduction‐oxidation mechanism.

### 2.5 Chemically Fueled Regenerative Chemiluminescence (CFRCL) With PBI

While the previous examples demonstrated the feasibility of RCL with PBI derivatives via a stepwise procedure, given the reversible nature of the process and considering that it occurs under the mild conditions, it is now possible to advance this concept toward a truly dissipative, chemically fueled light‐emitting system. To achieve this, the water‐soluble compound **4^4+^** was identified as best candidate for Chemically Fueled Regenerative Chemiluminescence (CFRCL). First, the use of water enables operation in a medium that is compatible with the redox reagents employed, sodium dithionite (which exhibits poor solubility in DMF) and hydrogen peroxide. Furthermore, conducting the process in an aqueous medium can pave the way for potential translation of this methodology to biologically relevant environments.

Experimentally, we leveraged the high oxidative stability of **4^4+^** by preparing a solution containing both the luminophore (1 mM, 0.5 mL) and the oxidant (H_2_O_2_, 1 M). The dissipative cycle was initiated by the serial injection of the reducing fuel (sodium dithionite, 0.1 M, 20 eq. per injection). Each injection triggered a burst of intense chemiluminescence with a duration of approximately 10 s (Figure 4a). In this framework, compound **4^4+^** acts as a “chemiluminescent catalyst” that mediates the exergonic reaction between H_2_O_2_ and dithionite, yielding light as a transient by‐product.

**FIGURE 4:**
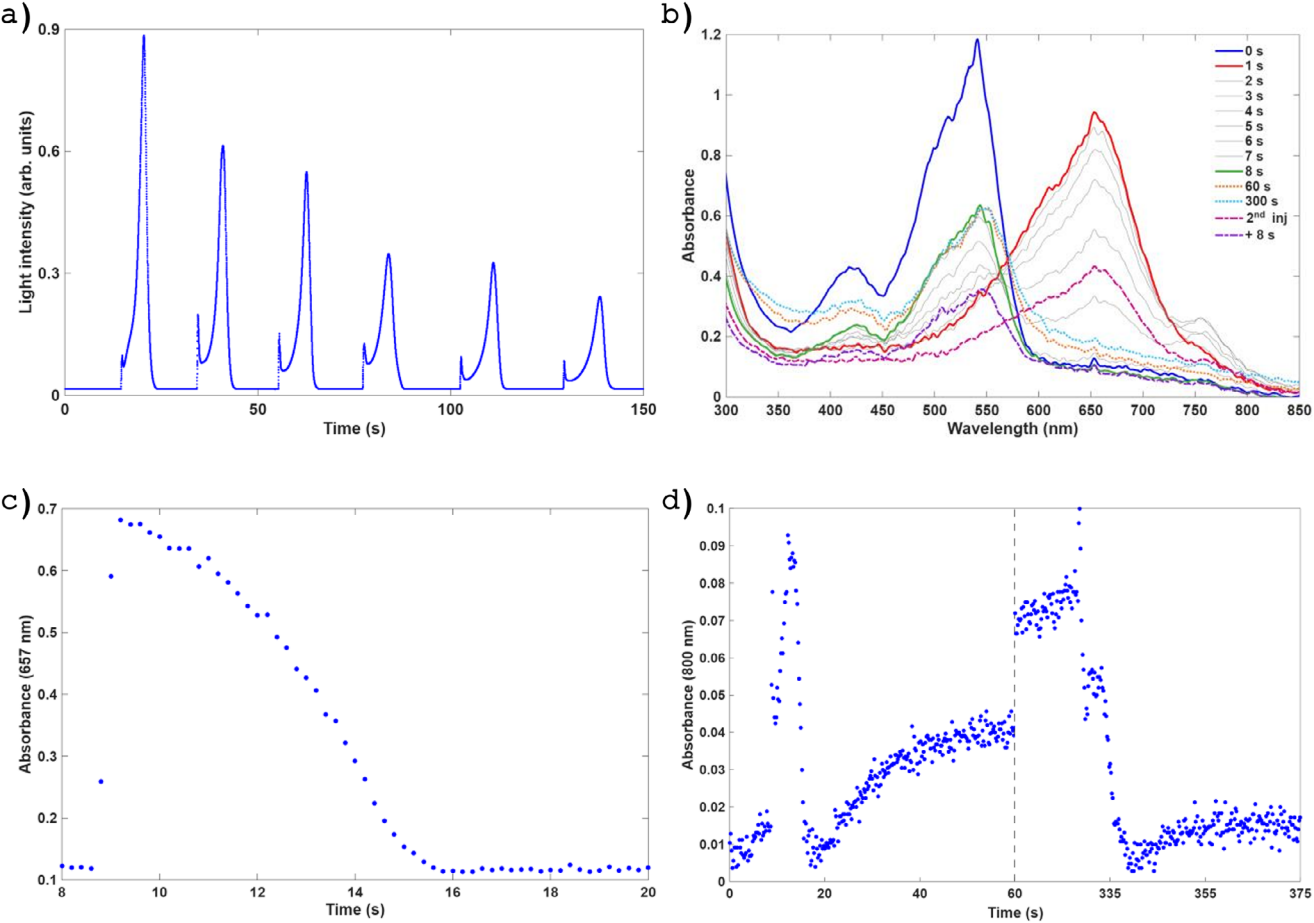
(a) Light intensity versus time profiles following multiple injections of 100 µL of a DTO solution (20 eq.) into a solution of **4** ^4+^ and H_2_O_2_ ([**4** ^4+^] = 1 mM, [H_2_O_2_] = 1 M, 0.5 mL); solvent: H_2_O. PMT gain 5. (b) Absorption spectra of compound **4** ^4+^ and H_2_O_2_ after addition of dithionite. Significant spectra: blue line ‐ 0 s; red line ‐ 3 s; green line ‐ 8 s; dashed orange line ‐ 60 s; dashed cyan line ‐ 300 s; purple line—second DTO injection; violet line ‐ 8 s after second DTO injection. (c) Time evolution of the absorbance of the doubly reduced intermediate **4** ^2+^ (657 nm) upon addition of DTO, recorded every 200 ms. (d) Time evolution of the absorbance at 800 nm to monitor scattering evolution within the system, recorded every 200 ms.

Mechanistically, this behavior suggests that **4** ^4+^ reacts preferentially with dithionite to form a reduced intermediate, which is subsequently oxidized by H_2_O_2_ with a driving force sufficient to populate the excited state **4^4+^** *. Monitoring the decomposition of DTO by UV–vis spectroscopy (*λ* = 352 nm) in the presence and absence of compound **4^4+^** revealed an enhanced rate of DTO consumption upon addition of the luminophore (Figure S21). This observation indicates that compound **4^4+^** actively participates in a reaction with DTO, competing with H_2_O_2_ for the same substrate. The resulting system sustains light emission as long as both chemical fuels are available, shifting the control of the process from thermodynamic equilibrium to kinetic regime. The observed decrease in light intensity and the broadening of the emission spikes during subsequent injections (Figure 4a) can be attributed to the cumulative dilution of the system (0.1 mL additions into an initial 0.5 mL volume, Figure S22 showing linear correlation). Subsequently, we examined the effect of the increase in ionic strength induced by the addition of DTO. Because of the relatively high luminophore concentrations employed, DTO injections can promote aggregation of compound **4^4+^**, leading to ACQ and thereby suppressing an efficient radiative deactivation pathway within the RCL system. To monitor ACQ arising from the supramolecular assembly of compound **4^4+^**, we measured the PLQY and recorded the emission spectra of the system after each addition of DTO. Notably, already after the first injection, the PLQY exhibited a pronounced decrease and rapidly approached approximately 5% (Figure S23). In parallel, the initially clear solution gradually transformed into a turbid suspension, and the emission spectra displayed a bathochromic shift accompanied by broadening (Figure S23), both of which are indicative of aggregation processes. Surprisingly, the CL emission spectrum of the CFRCL system recorded after the sixth DTO injection still coincides with the PL spectrum of molecularly dissolved compound **4^4+^** (Figure S24). Taken together, these observations imply that the reduction process disassembles the aggregates. Then, the highly reactive, molecularly dispersed reduced intermediates undergo fast oxidation by H_2_O_2_, generating the singlet excited state from which CL emission takes place. Because this chemiluminescent reaction pathway is orders of magnitude faster than the subsequent aggregation process, light emission occurs almost exclusively from the transiently disassembled, monomeric species. It is only after emitting light and relaxing back to the ground state that the dye molecules slowly undergo salt‐induced re‐aggregation (H‐type self‐assembly), which operates on a much slower timescale than chemiluminescence decay. A real‐time monitoring of the out‐of‐equilibrium system via UV–vis spectroscopy allows to identify the critical intermediates and to capture the formation and dissolution of the aggregates (Figure 4b). Upon DTO addition (50 µL, 0.1 M) to a solution of compound **4^4+^** (0.5 mM) and H_2_O_2_ (1 M), the absorbance at 657 nm (corresponding to the doubly reduced species **4** ^2+^) immediately spikes, indicating a rapid displacement from equilibrium. The system then autonomously relaxes back to its initial state as the intermediate is consumed by H_2_O_2_, regenerating the starting dye and terminating with light emission (Figure 4b,c). Crucially, the final absorbance relative to **4^4+^** is reduced by approximately 50% as a consequence of the twofold increase in solution volume (see Supporting Information for experimental details). Notably, a few seconds after the system is allowed to relax back to equilibrium, a scattering signal (monitored at 800 nm, a wavelength at which the involved species do not exhibit significant absorption) starts to develop. This behavior can be attributed to the slow formation of aggregates of compound **4^4+^**, driven by the increased salt concentration in solution. If the system is left undisturbed for 5 minutes, the scattering contribution increases steadily (Figures 4d and S25). However, upon a second addition of DTO, the equilibrium is again perturbed, leading to the reduction of compound **4^4+^** and concomitant dissolution of the aggregates. Similar phenomena have previously been reported for chemically fueled systems based on PBI derivatives [18]. Importantly, when the intermediate reduced species is subsequently reoxidized, no scattering is detected (Figure 4d), indicating that compound **4^4+^** is regenerated in a molecularly dissolved state. Nevertheless, after a few seconds, the increase in apparent absorbance at 800 nm reveals that aggregation of compound **4^4+^** resumes. The reduced scattering intensity observed after the second injection is again attributed to dilution of the system. If we repeat the experiment in more dilute condition (3 mL, **4^4+^** 0.01 mM, H_2_O_2_ 1 M), we obtain the same result except that the aggregation upon salt addition does not occur in the minutes time scale. (Figures S26, S27).

Key factors that compete in regulating the chemiluminescence output are the concentration and stoichiometric amount of the three components of the system: luminophore, reducing and oxidating agents. To study their influence, we employed a high‐throughput microplate reader setup. This approach ensured superior reproducibility and allowed for precise control over injection rates and mixing. The total photon emission (integrated area of the PMT signal) showed a clear positive linear dependence on both the luminophore concentration (from 0.1 to 1 mM, Figure 5a) and the oxidant concentration (H_2_O_2_ from 0.1 to 1 M, Figure 5b). These trends confirm that under these conditions, the light output is limited by the availability of the catalytic scaffold and the driving force of the oxidative step. However, a non‐linear relationship was observed when varying the concentration of the reducing agent (DTO, Figure 5c). This deviation can be rationalized by the kinetics of the underlying DTO‐H_2_O_2_ reaction, competing with the DTO‐luminophore one. Indeed, while this reaction is reported to follow a half‐order dependence on DTO under dilute (0.25–0.5 mM) and buffered conditions [35], when studied under concentration window of CFRCL, the UV‐Vis analysis reveals a different picture. Specifically, DTO decomposition rate increases by decreasing its concentration and keeping the concentration of H_2_O_2_ constant (Figure S28). This result is consistent with studies that report that high concentrations of DTO in unbuffered solution, the decomposition of DTO with hydrogen peroxide follows a much more complex path, leading to a deviation from simple elementary kinetics [36] (see Supporting Information for detailed discussion). As a result, in the CFRCL system, when lower concentrations of DTO are used, its direct reaction with H_2_O_2_ becomes faster and more competitive against the reaction between DTO and the luminophore, finally leading to a non‐linear CL output. These findings are crucial for the future design of CFRCL‐based analytical tools, where the chemical environment and ionic background must be carefully calibrated to ensure accurate signal interpretation.

**FIGURE 5:**
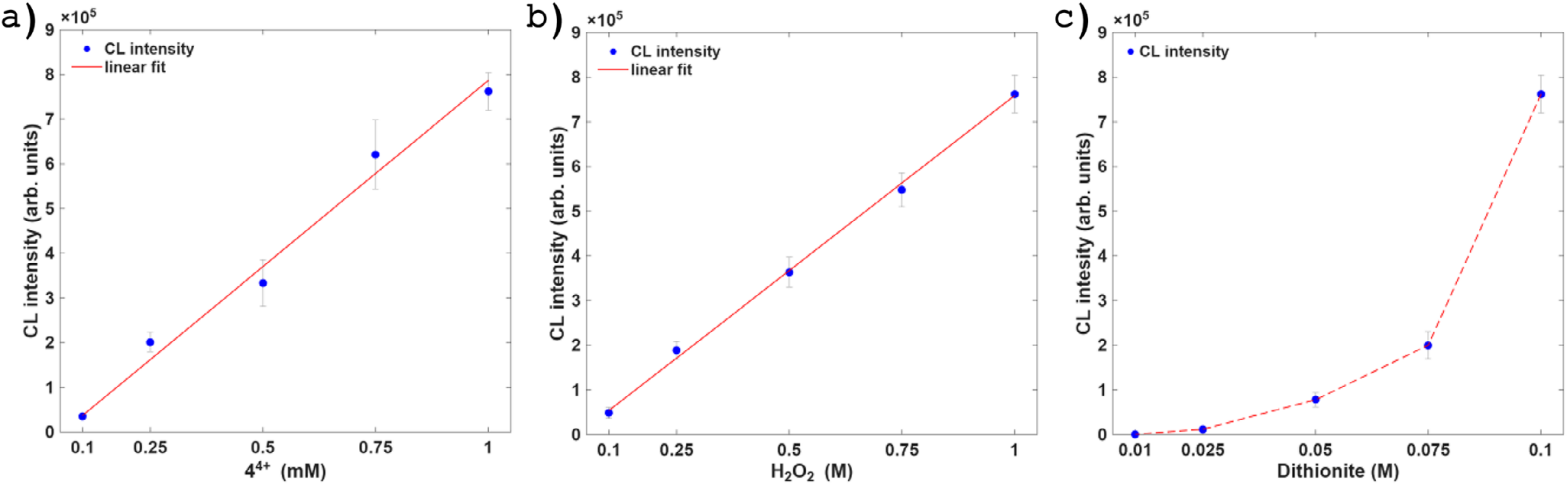
Concentration dependance of chemiluminescence intensity with the variation of the concentration of single components. (a) Dependance with luminophore. [**4** ^4+^] = 1, 0.75, 0.5, 0.25, 0.1 mM; [H_2_O_2_] = 1 M, [DTO] = 0.1 M, solvent: water. (b) Dependance with H_2_O_2_. [H_2_O_2_] = 1, 0.75, 0.5, 0.25, 0.1 M; [**4** ^4+^] = 1 mM, [DTO] = 0.1 M, solvent: water. (c) Dependance with dithionite. [DTO] = 0.1, 0.075, 0.05, 0.025, 0.01 M; [**4** ^4+^] = 1 mM, [H_2_O_2_] = 1 M, solvent: water; the dashed red line is a guide for the eye.

## 3 Conclusions

In summary, this work describes the rational development of a functional Regenerative Chemiluminescence (RCL) system operating through a mild reduction‐oxidation cycle in aqueous media. By strategically modifying the perylene scaffold, we transformed the commercially available compound **1**, which is inert to mild reduction, into a series of Perylene Bisimide (PBI) derivatives (**2**–**4** ^4+^) with finely tuned electrochemical and photophysical properties. Specifically, the incorporation of imide groups and bay‐functionalization shifted the reduction potentials to more anodic values, enabling the use of sodium dithionite as a mild chemical fuel.

Among the derivatives, compound **3** demonstrated high chemical robustness and efficient light emission in organic media, with the complete recovery of the neutral luminophore after each redox cycle. The successful translation of this architecture to the water‐soluble derivative **4^4+^** allowed us to achieve, for the first time, RCL in a fully aqueous environment under mild conditions. This represents a significant milestone toward the application of RCL in biological contexts.

The most transformative result of this study is the transition from a stepwise RCL process to a dissipative regime, which we have designated as Chemically Fueled Regenerative Chemiluminescence (CFRCL). In this out‐of‐equilibrium system, the PBI luminophore acts as a chemiluminescent catalyst, mediating the exergonic reaction between dithionite and H_2_O_2_ to generate sustained light emission. By operating in a dissipative state, we shifted the control of light production from thermodynamic to kinetic regime.

Our systematic investigation into the influence of component concentrations revealed a linear relationship between light output and the concentrations of both the luminophore and the oxidant (H_2_O_2_).

These findings open to the possibility of expanding this framework toward milder and biomimetic reducing agents, envisioning the potential of the CFRCL system for bio‐analytical, sensing, and imaging applications triggered by endogenous reductants and reactive oxygen species (ROS).

Overall, this work bridges the gap between classic electron‐transfer chemiluminescence and modern systems chemistry, providing a versatile platform for creating autonomous, non‐equilibrium light‐emitting materials and diagnostic tools.

## Author Contributions

**Luca Morgan**: methodology, data curation, investigation, visualization, writing – original draft, writing – review and editing. **Chiara Alberoni**: investigation, methodology, data curation, visualization, writing – original draft, writing – review and editing. **Dario Alessi**: methodology, data curation, investigation, formal analysis, supervision, visualization, writing – original draft, writing – review and editing. **Daniel Morales‐martinez**: methodology, data curation, investigation, validation, formal analysis, visualization, writing – original draft, writing – review and editing. **Sara Bonacchi**: investigation, supervision, formal analysis, writing – review and editing, writing – original draft. **Alessandro Aliprandi**: conceptualization, methodology, investigation, validation, formal analysis, supervision, funding acquisition, visualization, project administration, resources, writing – original draft, writing – review and editing, data curation.

## Conflicts of Interest

The authors declare no conflicts of interest.

## Supporting information




**Supporting File**: anie74474‐sup‐0001‐SuppMat.docx.

## Data Availability

The data that support the findings of this study are available in the supplementary material of this article
