# Supplementary material for: Dissipative Regenerative Chemiluminescence in Water: A Perylene‐Based Out‐of‐Equilibrium Light‐Emitting System
Source: Angew Chem Int Ed Engl. 2026 Aug 27;65(43):e9467229. doi: 10.1002/anie.9467229 (PMC13676405; doi:10.1002/anie.9467229)
Supplement: Supplementary file 1 [file ANIE-65-e9467229-s001.docx]

**Dissipative Regenerative Chemiluminescence in Water: A Perylene-Based Out-of-Equilibrium Light-Emitting System**

Luca Morgan, Chiara Alberoni, Dario Alessi, Daniel Morales-Martinez, Sara Bonacchi, Alessandro Aliprandi*

Dipartimento di Scienze Chimiche, Università Degli Studi di Padova, Via Marzolo 1, Padova 35131, Italy.

Supporting information

| **General methods** |  | **2** |
| --- | --- | --- |
| **Synthesis** |  | **5** |
| **Electrochemical characterization** |  | **8** |
| **Photophysical characterization** |  | **10** |
| **Chemiluminescence experiments** |  | **16** |
| **^1^H NMR and HR-MS** |  | **23** |
| **Table** |  | **28** |
| **Supplementary references** |  | **29** |

**General methods**

**Chemical**

All solvents and compounds were purchased from Sigma Aldritch and used as received.

^1^H, and ^13^C{^1^H} spectra were recorded on a Bruker AVANCE III spectrometer equipped with a BBO probe. ^1^H and ^13^C chemical shifts were referenced relative to residual solvents peaks. The ^1^H NMR chemical shifts (δ) are given in ppm and refer to residual protons on the corresponding deuterated solvent. All coupling constants (*J*) are given in Hertz (Hz). All deuterated solvents were used as received without any further purification.

Mass spectrometry analyses were performed with an ACQUITY UPLC I-Class PLUS System equipped with high-resolution RDa Time of-Flight (ToF) mass analyzer with an Electrospray Ionization (ESI) source (Waters Corporation).

**Electrochemical measurements**

Electrochemical measurements were performed with an Autolab PGstat302N as potentiostat using NOVA 2.1.5 as interface software. A three-electrode configuration with a glassy carbon electrode (diameter 3 mm) as working electrode (WE), a silver/silver chloride as a quasi-reference electrode (RE) built according to a literature procedure^1^ and a platinum wire as counter electrode (CE).

Before each experiment the working electrode was polished with alumina slurry (0.05 mm). The solution was Ar-bubbled for 5 minutes to remove oxygen, and each experiment was performed under argon atmosphere.

Cyclic voltammetry of compound **3** was performed in dimethylformamide solution containing 1 mM of investigated compound and 0.1 M tetrabutylammonium hexafluorophosphate (TBAPF_6_) as supporting electrolyte.

Cyclic voltammetry of compound **4^4+^** was performed in phosphate buffer solution (PBS) solution containing 1 mM of investigated compound.

**Photophysical measurements**

Absorption spectra were recorded with a Varian Cary 100bio UV-VIS spectrophotometer in 10 mm quartz cuvettes (Hellma GmbH & Co. KG). Emission spectra and absolute PhotoLuminescence Quantum Yields (PLQYs) were obtained with a Hamamatsu Absolute PL quantum yield spectrometer C11347 Quantaurus QY using a quartz tube. Photophysical measurements were performed with DMF or aqueous solutions containing 5·10^-6^ M of compounds investigated.

**Spectroelectrochemical measurements**

UV-Vis absorption spectroelectrochemical measurements were performed using a CHI760 potentiostat for the constant pulse electrolysis at a constant potential and an Ocean Optics Spectrophotometer with a QE65000 detector was used to follow the evolution of the absorption spectrum. A three-electrode electrochemical cell was used for the electrolysis experiments; a customized glassy carbon plate electrode with a split of ~0.3 mm was used as a working electrode to electrolyze the electroactive species and a platinum wire was the counter electrode and a silver wire separated in a salty bridge containing a saturated solution of tetrabutylammonium hexafluorophosphate (TBAPF_6_) was used as pseudo-reference electrode. The potentials were corrected using the formal potentials of the analyzed species obtained in the previous voltammetric characterization. The experiments with compound **3** (1mM) were performed using anhydrous DMF 99.8% as the solvent and 0.2 M TBAPF_6_ was used as the supporting electrolyte, while with compound **4**^4+^ (1 mM) the experiments were carried out in PBS solution. Before the experiments, the solutions were deoxygenated using argon bubbling. The experiments were performed under an argon atmosphere to avoid the presence of oxygen. The electrolysis was performed for 300 seconds (five minutes) in a customized cuvette of 2 mm for the electrolysis and the UV-Vis spectrum was registered after this time.

**Chemiluminescence measurements**

Chemiluminescence setup

A custom setup was developed to record chemiluminescence.

**Figure S1** Schematic representation of the custom setup for chemiluminescence measurements.

Chemiluminescence occurs upon mixing of two solutions: a first solution (solution 1) is injected with a syringe connected to a microfluidics Teflon tube into a second solution (solution 2) contained in a flat-bottomed vial. The Teflon tube is required since the vial and the recording setup are contained in a black box, while the syringe is kept outside. Before each experiment the Teflon tube is washed with water, the solvent used for the experiment and conditioned with the solution to be injected.

Two configurations are possible:

**PMT configuration.** The total amount of photons produced by the chemiluminescent system is recorded by placing the vial over a Thorlabs 1001/M photomultiplier tube. PMT 2100 control was used as software interface for photomultiplier tube. Alternatively, the vial can be connected to the PMT through an Ocean Insight 1 mm optical fiber.

**CCD configuration.** The chemiluminescence emission spectrum of the system is recorded by placing the vial over an Ocean Insight 1 mm optical fiber connected to a CCD QEPro camera (Ocean Insight). The CCD camera was set with a 200 μm slit.

• Chemiluminescence experiments with aqueous sodium dithionite as reducing agent and hydrogen peroxide as oxidant.

A solution of luminophore (1 mM) was prepared and degassed for 10 min by Ar-bubbling. Then, under Ar atmosphere, 1 mL of solution was transferred into a sealed vial and 100 μL of an aqueous solution of sodium dithionite (0.1 M, 10 eq.) was added. The resulting solution (solution 1) was taken with a syringe and 0.5 mL were flash injected into a H_2_O_2_ solution (1 M) (solution 2) contained in a flat-bottomed vial inside the black box. Both PMT and CCD configurations are used to collect the chemiluminescence emission.

Compounds **2** and **3** required DMF for both compound solubilization and preparation of the oxidant solution. With compound **4**^4+^ deionized water was used. All the solutions were freshly prepared.

• Chemically fueled regenerative chemiluminescence with aqueous sodium dithionite as reducing agent and hydrogen peroxide as oxidant (compound **4**^4+^).

A solution of compound **4**^4+^ and H_2_O_2_ (1 mM and 1 M in deionized water, respectively) is prepared and 0.5 mL of solution (solution 2) are inserted into a vial placed in the black box. Then subsequent multiple flash injections of 100 μL of sodium dithionite (0.1 M in deionized water, 20 equivalents) (solution 1) are performed. PMT and CCD configurations are adopted to collect the chemiluminescence emission in real time.

Study of the concentration of single components

A Flexstation 3 Multi-Mode Microplate Reader was used to perform CFRCL experiments and to monitor light emission with superior accuracy and reproducibility to study the effect of single components on light output of the system. A 384 well-plate with transparent bottom and black walls was used as support to conduct the CFRCL reactions. Similarly to CFRCL experiments previously described, light emission was monitored upon injection of a solution of reducing agent into a solution containing the luminophore and the oxidant. Compound **4**^4+^ was used as luminophore, sodium dithionite as reducing agent and hydrogen peroxide as oxidizing agent. Deionized water was used as solvent. Freshly prepared solutions were used. For each experiment 15 μL of compound solution and 15 μL of H_2_O_2_ solution were introduced in a well and homogenized by gently shaking the plate. The reducing solution was loaded in a separate plate. During the experiment, the instrument took 60 μL of reducing agent and injected them into the solution of luminophore and H_2_O_2_, while monitoring from beneath the well the evolution of light with a PMT. Concentrations of luminophore were varied between 1 mM, 0.75 mM, 0.50 mM, 0.25 mM and 0.01 mM, while keeping constant the concentration of H_2_O_2_ (1 M) and sodium dithionite (0.1 M). Concentrations of H_2_O_2_were varied between 1 M, 0.75 M, 0.50 M, 0.25 M and 0.01 M, while keeping constant the concentration of luminophore (1 mM) and sodium dithionite (0.1 M). Concentrations of sodium dithionite were varied between 0.1 M, 0.075 M, 0.050 M, 0.025 M and 0.001 M, while keeping constant the concentration of luminophore (1 mM) and H_2_O_2_ (1 M). Each experiment was performed in quadruplicate.

**UV-Vis absorption spectra of the out-of-equilibrium system**

UV-Vis absorption measurements of the out-of-equilibrium system were performed using a Avantes Avalight-DHS as light source in the UV-Vis range and an Ocean Optics Spectrophotometer with a CCD QEPro detector to follow the evolution of the absorption spectrum. A qpod 3 cuvette holder mounted with imaging lenses in the “absorption” configuration was used to align the setup and to stir the solution. The reactions with high concentration of compound **4^4+^** were performed in 1 mm quartz cuvettes (Lab Analytics), while reactions at lower concentrations of **4^4+^** were performed in 10 mm quartz cuvettes (Hellma GmbH & Co. KG). Experimentally, at high luminophore concentrations, 50 μL of an aqueous solution of compound **4**^4+^ (0.5 mM) and H_2_O_2_ (1 M) were inserted into the cuvette and placed in the qpod 3 holder. The recording of UV-Vis absorption spectra every 200 ms was started, then 50 uL of an aqueous solution of sodium dithionite (0.1 M) were injected into the cuvette to take the system out-of-equilibrium. The system was allowed to evolve unstirred for 5 minutes, then a second injection of dithionite (0.1 M, 100 μL) was performed. The system was monitored, unstirred, for other 5 minutes.

When working at lower luminophore concentrations, 3 mL an aqueous solution of compound **4**^4+^ (0.01 mM) and H_2_O_2_ (1 M) were inserted into the cuvette and placed in the qpod 3 holder. The recording of UV-Vis absorption spectra every 50 ms was started, then 100 uL of an aqueous solution of sodium dithionite (0.1 M) were injected into the cuvette to take the system out-of-equilibrium. A KF Technology NE-8060 syringe pump was used to inject the sodium dithionite solution into the cuvette.

**Synthesis**

*Synthesis of N,N’-Di(2,6-diisopropylphenyl)- 3,4,9,10-tetracarboxylic acid bisimide^2^ (****2****)*

Perylene-3,4,9,10-tetracarboxylic dianhydride (200 mg, 0.38 mmol), 2,6-diisopropylaniline (2.2 eq., 147.2 mg, 0.83 mmol) and imidazole (2 g) were introduced in a round flask and stirred under inert atmosphere for 16 h at 140 °C. Then, 40 mL of an aqueous solution of HCl (2 M) was added to the reaction mixture. An orange solid precipitated at the suspension was stirred at room temperature for 30 minutes. The solid was filtered, washed with water and EtOH. The product was obtained as an orange powder (151.6 mg, 57 %).

^1^H NMR (400 MHz, CDCl_3_, 298 K): δ (ppm) = 8.96 – 8.61 (m, 16H), 7.38 (d, ^3^*J*_HH_ = 7.9 Hz, 4H), 1.20 (d, ^3^*J*_HH_ = 6.8 Hz, 24H) (CH *i*Pr signal too weak).

*Synthesis of 2,5,8,11-Tetrachloroperylenetetracarboxylic acid dianhydride^3^*

A mixture of perylene-3,4,9,10-tetracarboxylic dianhydride (5.00 g, 12.7 mmol) and iodine (0.54 g, 2.12 mmol) in chlorosulfonic acid (20 mL) was stirred at 65 °C for 20 h. The hot solution was filtered and the obtained solution was added dropwise into ice cold water with stirring and the resulting orange precipitate was filtered, washed with water and dried under vacuum. It was obtained as an orange solid in a high yield (6.70 g, 99 %).

^1^H NMR (300 MHz, CDCl_3_), δ 8.75 (s, H^pery^).

*Synthesis of N,N’-Di(2,6-diisopropylphenyl)-1,6,7,12-tetrachloroperylene-3,4,9,10-tetracarboxylic acid bisimide^4^*

A suspension of **1** (1.0 g, 1.88 mmol) and 2,6-diisopropylaniline (5 eq., 9.40 mmol) in 15 ml DMF was stirred under reflux for 48 h. The solvent was reduced and the dark red solid was collected, washed with water and dried. It was obtained in good yield (75 %) and used for further steps without purification.

^1^H NMR (CDCl_3_, 300 MHz) δ 8.78 (s, 4H, H^pery^), 7.56-7.51 (m, 2H, H^p^), 7.38 (d, J = 7.7 Hz, 4H, H^m^), 2.76 (sept, 4H, CH^iPr^), 1.20 (dd, J = 6.8 Hz, J = 3.2 Hz, 24H, CH_3_^iPr^).

*Synthesis of N,N’-Di(2,6-diisopropylphenyl)-1,6,7,12-tetra(3-pyridoxy)-perylene-3,4,9,10-tetracarboxylic acid bisimide^5^ (****3****)*

Compound **2** (500 mg, 0.59 mmol), 3-hydroxypyridine (8 eq., 4.70 mmol) and anhydrous K_2_CO_3_ (4 eq., 2.35 mmol) were added to DMF (30 mL). The solution was stirred at 80°C under argon atmosphere overnight. After cooling the mixture to room temperature and pouring into aqueous hydrochloride acid (50 mL, 1M), the precipitated product was filtered, centrifugated and washed several times with water and dried. The product **3** was a red solid obtained in good yields (73 %).

HR-MS m/z [M + H]^+^ calcd. 1083.4076; found 1083.4056. ^1^H NMR (400 MHz, CDCl_3_, 298 K): δ (ppm) = 8.42 (d, ^3^*J*_HH_ = 4.6 Hz, 4H), 8.36 (d, ^3^*J*_HH_ = 2.7 Hz, 4H), 8.30 (s, 4H), 7.55 – 7.44 (m, 2H), 7.39 (ddd, *J*_HH_ = 8.4, 2.8, 1.4 Hz, 4H), 7.35 – 7.30 (m, 8H), 2.71 (h, ^3^*J*_HH_ = 7.1 Hz, 4H), 1.15 (d, ^3^J_HH_ = 6.7 Hz, 24H). ^13^C{^1^H} NMR (101.4 MHz, CDCl3, 298 K): δ = 162.7, 155.2, 152.4, 145.7, 145.5, 141.0, 135.9, 133.3, 130.2, 129.9, 127.5, 125.0, 124.2, 124.0, 121.6, 121.1, 120.9, 29.3, 24.2 Hz.

*Synthesis of N,N’-Di(2,6-diisopropylphenyl)-1,6,7,12-tetra(3-pyridoxy)-perylene-3,4,9,10-tetracarboxylic acid bisimide^5^ (****4***^4+^*)*

An acetonitrile solution (15 mL) of **3** (150 mg, 0.14 mmol) was prepared and heated at 70°C. Methyl iodide (5.0 eq., 0.70 mmol) was then added by syringe with vigorous stirring. After 3 days, the solvent was evaporated under vacuum and **4**^4+^’ was dried. It was obtained in high yield (81 %). Perylene derivative with Cl^-^ (**4**^4+^) as counterion was obtained after stirring the compound with I^-^ for 3 days at room temperature using ion exchange resins.

HR-MS m/z [M^4+^] calcd. 285.6230; found 285.6248. ^1^H NMR (400 MHz, CD_3_OD, 298 K): δ (ppm) = 9.05 (d, 3JHH = 2.1 Hz, 4H), 8.67 (d, 3JHH = 6.0 Hz, 4H), 8.45 (d, ^3^*J*_HH_ = 11.4 Hz, 8H), 8.05 (dd, ^3^*J*_HH_ = 8.8, 5.9 Hz, 4H), 7.52 – 7.39 (m, 2H), 7.31 (d, ^3^*J*_HH_ = 7.7 Hz, 4H), 4.42 (s, 12H), 2.84 (p, ^3^J_HH_ = 6.7 Hz, 4H), 1.09 (d, ^3^*J*_HH_ = 6.8 Hz, 24H). ^13^C{^1^H} NMR (101.4 MHz, CD_3_OD, 298 K): δ = 163.3, 159.9, 155.3, 150.6, 145.9, 145.2, 141.1, 133.2, 129.5, 128.0, 125.2, 124.4, 123.9, 123.4, 121.3, 120.4, 29.1, 23.0 Hz. (CH3-py overlapped with solvent signal).

Ion-exchange was confirmed by electrochemical analyses. In the cyclic voltammogram of **4^4+’^**, two irreversible anodic peaks are observed at + 0.51 and + 0.94 V (vs Ag/AgCl), which are assigned to the stepwise oxidation of I^−^ to I_3_^−^ and of I_3_^−^ to I_2_, respectively.^6^ This assignment was validated by recording the cyclic voltammogram of methylpyridinium iodide (MePy^+^I^−^), in which the same oxidation features appear at identical potentials. In contrast, these anodic signals are absent in the cyclic voltammogram of compound **4^4+^**, thereby demonstrating the complete absence of iodide in this system. (Figure S4).

**Electrochemical characterization**

**Figure S2.** Cyclic voltammetry of **3**, 1 mM in DMF, 0.1 M TBAPF_6_, on glassy carbon = 3 mm. Scan rate = 100 mV s^-1^.

**Figure S3.** Cyclic voltammetry of **4**^4+^, 1 mM in PBS, on glassy carbon = 3 mm. Scan rate = 100 mV s^-1^.

**Figure S4.** Left) Cyclic voltammetries of methylpyridinium iodide (top), **4^4+^’** (center) and **4^4+^** (bottom). Right) Zoom of the anodic region. [compound] = 1 mM in DMF, 0.1 M TBAPF_6_, on glassy carbon = 3 mm. Scan rate = 100 mV s^-1^.

**Photophysical characterization**

**UV-Vis absorption spectra**

**Figure S5.** UV-Vis absorption spectrum of **1** (5·10^-6^ M in DMF).

**Figure S6.** UV-Vis absorption spectrum of **2** (5·10^-6^ M in DMF).

**Figure S7.** UV-Vis absorption spectrum of **3** (5·10^-6^ M in DMF).

**Figure S8.** UV-Vis absorption spectrum of **4**^4+^ (5·10^-6^ M in H_2_O·10^-6^ M in DMF).

**Emission spectra**

**Figure S9.** Normalized photoluminescence spectrum of **1** (5·10^-6^ M in DMF).

**Figure S10.** Normalized photoluminescence spectrum of **2** (5·10^-6^ M in DMF).

**Figure S11.** Normalized photoluminescence spectrum of **3** (5·10^-6^ M in DMF).

**Figure S12.** Normalized photoluminescence spectrum of **4**^4+^ (5·10^-6^ M in H_2_O).

**Figure S13.** PLQY and normalized photoluminescence of **3** (5·10^-6^ M) in different DMF/H_2_O ratios.

**Figure S14.** PLQY and normalized photoluminescence of **4**^4+^ (5·10^-6^ M) in different DMF/H_2_O ratios.

**Figure S15.** Absorption spectra of **1** (blue curve) and upon addition of sodium dithionite (100 eq.) (orange curve). [**1**] = 5·10^-6^ M in DMF.

**Figure S16.** Absorption spectra of **2** (blue curve), reduced **2** (with 100 eq. of sodium dithionite) (orange curve), **2** upon addition of H_2_O_2_ (yellow curve). [**2**] = 5·10^-6^ M in DMF.

**Figure S17.** stack of ^1^H NMR spectra (400 MHz, CDCl_3_, 298 K) of **2** pre (green traces, top) and post chemiluminescence experiment (red traces, bottom).

**Chemiluminescence experiments**

**PMT vs time profiles (stepwise CL)**

**Figure S18.** Light intensity vs time profile of **2**. [**2**] = 1 mM, 20 eq. of sodium dithionite, [H_2_O_2_] = 1 M, solvent: DMF. PMT (optical fiber) gain 100.

**Figure S19.** Light intensity vs time profile of **2**. [**2**] = 1·10^-5^ mM, 20 eq. of sodium dithionite, [H_2_O_2_] = 1 M, solvent: DMF. PMT (optical fiber) gain 100.

**Figure S20.** Absorption and emission spectra of compound **3** in DMF at different concentrations (0.005 mM, 0.01 mM, 0.1 mM and 1 mM). The emission spectra exhibit a large red shift of the maxima (from 602 to 621 nm) and a modification of the relative intensities of the peak and its shoulder due to increasing absorption onset at higher concentrations.

**Figure S21.** Variation of the absorbance at 352 nm monitoring the depletion of DTO (0.05 M, 3 mL in water) upon addition of H_2_O_2_ (10 M, 30 μL in water) in absence and in presence (3 mM in 30 μL) of compound **4^4+^**. The acquired data have been reported and analyzed as the function ΔA = (A(t)-Aꝏ)/(A_0_-Aꝏ) to compensate for the different final value of absorption at 352 nm due to the presence of compound **4^4+^**. The rate of DTO consumption within the system was estimated as the slope of the ΔA vs time curves considering the time needed to reach half of the initial amount of DTO. Transient increases in absorbance are attributed to light scattering by precipitated sulfur, which is generated when DTO oxidation is conducted under conditions of only limited excess hydrogen peroxide H_2_O_2_.^7^

**Figure S22.** Plot of the light intensity of each spike corrected for the dilution factor (V_final_/V_initial_) after every injection for the CFRCL of compound **4**^4+^ in H_2_O_2_ solution with dithionite ([**4**^4+^] = 1 mM, [H_2_O_2_] = 1 M; 0.5 mL; injections of 100 μL of a dithionite solution (20 eq.)).

**Figure S23.** PLQY and photoluminescence of the system in CFRCL conditions after each DTO injection. Initial PLQY is lower than the datum recorded in pure water due to dynamic quenching by H_2_O_2_.

**Figure S24.** Comparison of the emission spectra of **4**^4+^: photoluminescence (blue curve), regenerative chemiluminescence after the 6^th^ injection of DTO (red curve).

**Figure S25.** Time evolution of the absorbance at 800 nm to monitor scattering evolution within the CFRCL system, recorded every 200 ms. [**4^4+^**] = 0.5 mM, [H_2_O_2_] = 1 M, injections of [DTO] = 0.1 M: 50 μL, 100 μL.

**Figure S26.** Absorption spectra of compound **4**^4+^ and H_2_O_2_ after addition of dithionite. Significant spectra: blue line - 0 s; red line - 3 s; green line - 8 s after second DTO injection. [**4^4+^**] = 0.01 mM, [H_2_O_2_] = 1 M, injections of [DTO] = 0.1 M, 100 μL.

**Figure S27.** Time evolution of the absorbance of the doubly reduced intermediate **4**^2+^ (657 nm) upon addition of DTO, recorded every 50 ms. [**4^4+^**] = 0.01 mM, [H_2_O_2_] = 1 M, injections of [DTO] = 0.1 M, 100 μL.

**Figure S28.** UV–Vis absorbance decay of DTO (352 nm) with different initial concentrations of DTO and H_2_O_2_. To compare the curves at different DTO concentrations, data are reports as ΔA = (A(t)-Aꝏ)/(A_0_-Aꝏ).

Under the operating conditions employed in the CFRCL system, the reactions involving DTO, H_2_O_2_, and compound **4^4+^** proceed extremely rapidly, precluding a detailed kinetic characterization of the overall system in situ.

The kinetics of the isolated DTO-H_2_O_2_ reaction have been previously reported,^8^ and the following the rate law was established

$$-\frac{d[S_{2}O_{4}^{2-}]}{dt}=k\left[ H_{2}O_{2} \right]{[S_{2}O_{4}^{2-}]}^{1/2}$$

with k = 0.096 M^−1/2^s^−1^ (pH 6.5, phosphate-buffered). Critically, this half-order dependence on DTO concentration was determined under dilute, buffered conditions ([DTO] = 0.5-0.25 mM), nearly three orders of magnitude lower than the DTO concentrations employed in the present work for CFRCL (0.01-0.1 M).

We therefore investigated the DTO-H_2_O_2_ reaction independently by absorption spectroscopy, monitoring the decay of the DTO, under DTO concentrations comparable to those used in the CFRCL system. Experimentally aqueous solutions of DTO and H_2_O_2_ were freshly prepared before the analysis. DTO concentrations have been varied between 0.05 M, 0.025 M and 0.0125 M. H_2_O_2_ concentrations have been varied between 10 M and 5 M. In the quartz cuvette 3 mL of DTO solution were put under stirring and 30 μL of H_2_O_2_ solution were injected with a micropipette. Such ratio between DTO and H_2_O_2_ was necessary to prevent the reaction to be instantaneous and to allow to monitor the disappearance of DTO. However, under limited excess of H_2_O_2_, elementar sulfur can be precipitate^7^, causing momentaneous spikes on absorbance (Figure S28, blue line). DTO concentration was monitored by absorption at 352 nm with a frequency of 0.1 s. The reaction has been investigated under unbuffered conditions, since a buffered solution cannot be employed in the CFRCL system. The presence of salts, indeed, would induce the aggregation of compound **4^4+^**, making it unpractical to work with. We highlight, however, that under our experimental conditions ([DTO] > 0.01 M and unbuffered) the DTO-H_2_O_2_ system departs substantially from the simple regime described above. Kovács and Rábai^7^ have shown that, in unbuffered aqueous solution, the oxidation of dithionite ([DTO] = 3.75 mM) by H_2_O_2_ proceeds with a much more complex behavior. They reported several consecutive stages comprising transient accumulation of a SO_3_^2−^/HSO_3_^−^ buffer system, followed by autocatalytic oxidation of HSO_3_^−^ to SO_4_^2−^, with concomitant formation of S_2_O_6_^2−^. Throughout the course of the reaction, pH drift and oscillations are established, which modulate the rates of the numerous elementary steps responsible for DTO decomposition.

To compensate the different absorptions due to different DTO concentrations, the acquired data have been reported and analyzed as the function ΔA = (A(t)-Aꝏ)/(A_0_-Aꝏ) for a clearer comparison (Figure S28). The rate of DTO consumption within the system was estimated as the slope of the ΔA vs time curves considering the time needed to reach half of the initial amount of DTO.

Interestingly, lowering the DTO concentration from 0.05 M to 0.025 M and to 0.0125 M resulted in an increase, rather than a decrease, in the observed rate of DTO decomposition, with an apparent inverse order dependence on DTO. As a result, by lowering DTO concentrations its reaction with H_2_O_2_ appears to occur faster, thus in our system, under CFRCL conditions, by lowering the concentration of DTO, the reaction between DTO and H_2_O_2_ becomes more competitive with the reaction of DTO and the luminophore, thus resulting in a lower and non-linear response of light production. We emphasize, however, that under our experimental conditions ([DTO] > 0.01 M and unbuffered), the overall rate of DTO disappearance cannot be assumed to be governed by a single elementary step, and the apparent kinetic order with respect to DTO must be interpreted as a property of a complex system rather than the order of an elementary reaction.

**Figure S29.** Photos under white and UV light of the investigated compounds at each RCL cycle step.

**^1^H NMR**

**Figure S30** ^1^H NMR spectrum (400 MHz) of **2** in CDCl_3_ at 298 K.

**Figure S31** ^1^H NMR spectrum (400 MHz) of 2,5,8,11-tetrachloroperylenetetracarboxylic acid dianhydride in CDCl_3_ at 298 K.

**Figure S32** ^1^H NMR spectrum (400 MHz) of N,N’-Di(2,6-diisopropylphenyl)-1,6,7,12-tetrachloroperylene-3,4,9,10-tetracarboxylic acid bisimide in CDCl_3_ at 298 K.

**Figure S33** ^1^H NMR spectrum (400 MHz) of **3** in CDCl_3_ at 298 K.

**Figure S34** ^13^C{^1^H} NMR spectrum (101.4 MHz) of **3** in CDCl_3_ at 298 K.

**Figure S35** ^1^H NMR spectrum (400 MHz) of **4**^4+^ in MeOD-*d_4_* at 298 K.

**Figure S36** ^13^C{^1^H} NMR spectrum (101.4 MHz) of **4**^4+^ in MeOD-*d_4_* at 298 K.

**Figure S37.** HRMS of compound **3** (positive mode).

**Figure S38.** HRMS of compound **4^4+^** (positive mode).

| **Salts** | **PLQY (%)** |
| --- | --- |
| - - - | 44 |
| PBS | 67 |
| Na_2_SO_4_ (1M) | 67 |

**Table S1** Photoluminescence quantum yields of compound **4**^4+^ (5·10^-6^ M in H_2_O) with different concentrations of salts.

**Supplementary references**

[1] Thomas, J. M. Student Construction of a Gel-Filled Ag/AgCl Reference Electrode for Use in a Potentiometric Titration. J. Chem. Ed. 76, 97–98 (1999).

[2] Rosso, C., Williams, J. D., Filippini, G., Prato, M. & Kappe, C. O. Visible-Light-Mediated Iodoperfluoroalkylation of Alkenes in Flow and Its Application to the Synthesis of a Key Fulvestrant Intermediate. *Org Lett* **21**, 5341–5345 (2019).

[3] Partridge, B. E. *et al.* Increasing 3D supramolecular order by decreasing molecular order. A comparative study of helical assemblies of dendronized nonchlorinated and tetrachlorinated perylene bisimides. *J Am Chem Soc* **137**, 5210–5224 (2015).

[4] Qu, J., Kohl, C., Pottek, M. & Müllen, K. Ionic perylenetetracarboxdiimides: Highly fluorescent and water-soluble dyes for biolabeling. *Angewandte Chemie - International Edition* **43**, 1528–1531 (2004).

[5] Kohl, C., Weil, T., Qu, J. & Müllen, K. Towards highly fluorescent and water-soluble perylene dyes. *Chemistry - A European Journal* **10**, 5297–5310 (2004).

[6] Yaralyev, Y. A., Oxidation of iodide ions by means of cyclic voltammetry *Electrochimica Acta*, **29**, 1213-1214 (1984).

[7] Kovács, K. M.; Rábai, G. Large Amplitude pH Oscillations in the Hydrogen Peroxide-Dithionite Reaction in a Flow Reactor *J. Phys. Chem. A*, **105**, 9183–9187 (2001).

[8] Creutz, C.; Sutin, N. Kinetics of the Reactions of Sodium Dithionite with Dioxygen and Hydrogen Peroxide *Inorganic Chemistry*, **13**, 2041–2043 (1974).
